# Bilateral Morgagni Hernia: A Unique Presentation of a Rare Pathology

**DOI:** 10.1155/2016/7505329

**Published:** 2016-06-14

**Authors:** Michael Leshen, Randy Richardson

**Affiliations:** Creighton University School of Medicine, Phoenix Regional Campus, 350 W. Thomas Road, Phoenix, AZ 85013, USA

## Abstract

Morgagni hernia is an unusual congenital herniation of abdominal content through the triangular parasternal gaps of the anterior diaphragm. They are commonly asymptomatic and right-sided. We present a case of a bilateral Morgagni hernia resulting in delayed growth in a 10-month-old boy. The presentation was unique due to its bilateral nature and its symptomatic compression of the mediastinum. Diagnosis was made by 3D reconstructed CT angiogram. The patient underwent medical optimization until he was safely able to tolerate laparoscopic surgical repair of his hernia. Upon laparoscopy, the CT findings were confirmed and the hernia was repaired.

## 1. Introduction

Morgagni hernias are a rare finding that represent roughly 2% of all congenital diaphragmatic hernias [1]. They occur when abdominal content herniates through triangular parasternal gaps [2]. They typically occur on the right side of the sternum, though they can rarely occur on the left or bilaterally [3]. They are generally asymptomatic and incidentally found during an unrelated diagnostic workup [4]. We present an unusual case of bilateral Morgagni hernias with focal liver herniation associated with an atrial-septal defect and right ventricular hypertrophy.

## 2. Case Presentation

A 10-month-old boy presented with poor weight gain. His parents noted he had been having some difficulty breathing and they were concerned about his activity level. Physical exam showed a child in moderate distress with mild pectus excavatum and a soft systolic murmur best heard at the left sternal border. The patient's murmur prompted an echocardiogram, which revealed a large atrial-septal defect (ASD) with 4-chamber cardiac enlargement. However, the cardiac enlargement was out of proportion to the expected volume overload of the patient's ASD. The patient was placed on 6 mg of furosemide twice a day until further workup and treatment could be obtained.

**Figure 1:**
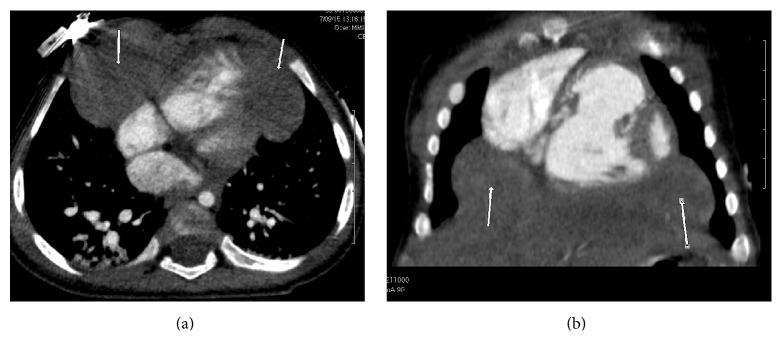
Axial (a) and coronal (b) images from a cardiac CTA of the chest show focal, bilateral anteromedial herniation of liver (arrows).

**Figure 2:**
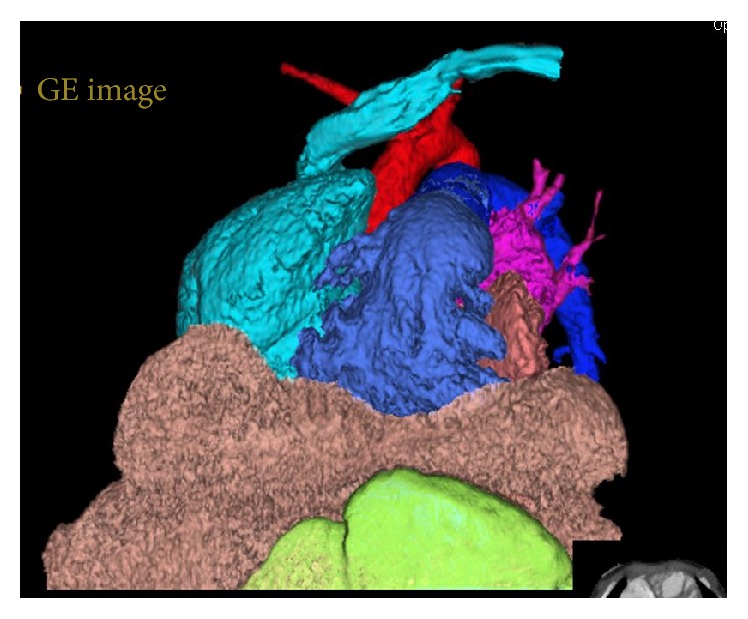
3D color coded cardiac CTA frontal projection shows bilateral focal liver (tan) herniating up through the anteromedial aspect of the diaphragm in front of the right atrium (purple) and right atrium (light blue).

Cardiac CT was obtained in order to identify the etiology of the disproportionate cardiac enlargement. The CT did not show any additional anatomic abnormalities of the heart but revealed large bilateral anterior diaphragmatic hernias with a large portion of the liver in the chest and crowding the right ventricle (Figure 1). A 3D reconstruction of the hernia was generated to help better visualize the anatomy prior to surgery (Figure 2). The hernia was confirmed and repaired with a laparoscopic approach. The hernia was visualized and the liver was pulled back into the abdomen. The defect was then repaired with mesh. The patient is currently recovering from his Morgagni hernia repair and will have his ASD repaired at some point in the future.

## 3. Discussion

Giovanni Battista Morgagni, an Italian anatomist, first described the herniation of abdominal contents through the sternochondral triangles in 1769 based on cadaver observation [5]. Later, in 1828, Larrey described a surgical approach to the pericardial sac through these same triangles [6]. Due to their early work, the costochondral triangles have been referred to as the foramen of Morgagni on the right and the space of Larrey on the left, though the literature is inconsistent. They form when the pars sternalis and a costochondral arch fuse and close around the internal thoracic artery as it becomes the superior epigastric artery. Occasionally these spaces do not fully close and allow for the herniation of abdominal contents into the thorax. When this occurs, it is referred to as a Morgagni hernia regardless of laterality [2, 7].

Our patient presented with a rare bilateral Morgagni hernia. This presentation only makes up 2% of all Morgagni hernias. The majority of these hernias, 90%, occur on the right side of the sternum while the remaining 8% are present on the left [8]. Morgagni hernias are typically asymptomatic and are incidentally discovered during unrelated workups. When they are symptomatic, they usually present as respiratory or gastrointestinal complaints [9]. The hernias most frequently contain omental fat and transverse colon. They rarely contain liver, such as our case, or stomach [10]. Morgagni hernias can be diagnosed during any period of life including prenatal period [11].

Radiographic diagnosis of diaphragmatic hernias is typically made with chest radiographs, ultrasound (US), or computed tomography (CT). Chest radiography often requires anterior-posterior images to evaluate hernia severity and lateral images to evaluate hernia location. Images can vary depending on the contents of the hernia. Solid viscera protruding through the hernia may show an opaque hemithorax with or without mediastinal shift. Hollow viscera are often present as loops of bowel within the thorax. Air can be introduced into the bowel via a nasogastric tube if the content of the hernia is unclear. If the bowel is present in the hernia, the air will inflate and demonstrate loops of bowel in the thorax. An anterior medial mass on chest radiography can be suggestive of a Morgagni hernia. However, a differential diagnosis to consider would include pneumonia, atelectasis, diaphragmatic eventration, mediastinal lipoma, liposarcoma, abscess, and pleuropericardial cyst [12].

Computed tomography or ultrasound can be used to confirm a suspected diaphragmatic hernia. Multiphase CT can demonstrate the diaphragm and the organs that herniate through it. Omental vessels can be visualized in some diaphragmatic hernias and can help differentiate it from lipomas or liposarcomas. Intravenous contrast can help enhance these vessels and confirm a diagnosis [10].

Ultrasound is helpful in assessing diaphragmatic hernias that contain solid viscera. Hepatic echo-texture and color Doppler sonogram can confirm liver in thorax [13]. Hernias that contain hollow viscera can be more difficult to evaluate with US. Air in the bowels and lungs, as well as rib shadowing, can often distort the images and make them difficult to interpret [10].

Before the 1980s, every symptomatic congenital diaphragmatic hernia was treated as an emergency. However, that approach has shifted towards delayed surgery when it was demonstrated that repair of congenital diaphragmatic hernias significantly diminishes respiratory function postoperatively [14, 15]. Hernias involving intestinal obstruction, volvulus, or any other potentially lethal complications are still treated emergently. Those that are asymptomatic and incidentally discovered are also recommended for surgical repair, though there is no consensus regarding a time frame [16].

In summary, we present a child with a bilateral Morgagni hernia and cardiac enlargement due to an ASD. It is assumed that the worsening cardiac enlargement was due to the patient's Morgagni hernia, though this cannot be confirmed without patient follow-up. This case is unique in that it demonstrates a rare presentation of a rare pathology. A literature search for Morgagni hernias reveals several case reports throughout the years with varying patient ages and presentations. To our knowledge, this is the first report of a Morgagni hernia worsening cardiac enlargement due to an ASD in a child.
